# Delayed post-polypectomy bleeding following cold snare polypectomy of lesions <10 mm in patients on high-dose antithrombotic therapy: insights from a Dutch colonoscopy cohort

**DOI:** 10.1055/a-2721-3151

**Published:** 2025-11-20

**Authors:** Querijn N. E. van Bokhorst, Sophie te Marvelde, Jos W. Borkent, Paul Fockens, Evelien Dekker, Manon van der Vlugt

**Affiliations:** 1 Department of Gastroenterology and Hepatology, Amsterdam UMC, Amsterdam, The Netherlands; 2 Amsterdam Gastroenterology Endocrinology Metabolism, Amsterdam, The Netherlands; 3 Cancer Center, Amsterdam, The Netherlands; 4 6032 Lectorate for Nutrition, Dietetics and Lifestyle, HAN University of Applied Sciences, Nijmegen, The Netherlands; 5 3718 Department of Gastroenterology, Waikato Hospital, Hamilton, New Zealand; 6 Department of Gastroenterology, Bergman Clinics, Amsterdam, The Netherlands

## Abstract

**Background:**

Current guidelines state that low-risk polypectomies (cold snare polypectomies of lesions <10 mm) can be safely performed with continuation of single antiplatelet therapy (low-dose antithrombotic therapy [ATT]). The safety of low-risk polypectomies with continuation of an anticoagulant or dual antiplatelet therapy (high-dose ATT) is uncertain.

**Methods:**

Data from 31 325 colonoscopies performed at two Dutch endoscopy centers were analyzed. The centers followed different protocols for the management of ATT around colonoscopies. Incidence of delayed post-polypectomy bleeding (DPPB) and thromboembolic events with continuation or discontinuation of different ATTs around colonoscopy were analyzed.

**Results:**

The overall incidence of DPPB for colonoscopies with only low-risk polypectomies was 11/12 291 (0.09%). The incidence of DPPB was similar for patients continuing either high- or low-dose ATT (0.58% vs. 0.30%; P = 0.61). Although the incidence of DPPB significantly differed between patients on continued high-dose and no ATT (0.58% vs. 0.07%; P = 0.04), the absolute risk difference was small (0.67%) and the number of patients required to discontinue high-dose ATT to prevent one case of DPPB was estimated at 150. The severity of DPPBs was comparable between all groups. The incidence of thromboembolic events with ATT discontinuation was 2/1098 (0.18%).

**Conclusion:**

The risk of DPPB after low-risk polypectomies was similar for patients who continued high- and low-dose ATT. Although higher compared with patients without ATT, the incidence of DPPB with continued high-dose ATT remains very low. Therefore, we suggest continuation of high-dose ATT for colonoscopy indications with a low risk of detecting advanced polyps.

## Abstract (de)

**Graphical Abstract:**

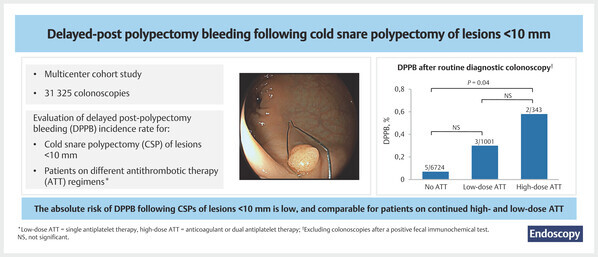

## Introduction

Removal of precancerous colorectal polyps (polypectomy) through colonoscopy is effective for prevention of colorectal cancer 1 2 . However, polypectomy is associated with a small risk of adverse events. Polypectomy-related bleeding, which can be divided into immediate post-polypectomy bleeding (IPPB) and delayed post-polypectomy bleeding (DPPB), represents the most common polypectomy-related adverse event 3 . IPPB is bleeding that occurs immediately after polypectomy, whereas DPPB occurs hours to days after colonoscopy. As immediate treatment of the bleeding source is usually not possible for DPPB, the clinical consequences are usually more significant than those of IPPB 4 .

The use of antithrombotic therapy (ATT) may increase the risk of DPPB 5 6 . Therefore, appropriate management of ATT around endoscopic procedures is required. Current guidelines classify polypectomy as an endoscopic procedure with a high bleeding risk and recommend discontinuation of all types of ATT except for aspirin monotherapy when polypectomy is performed 7 8 9 10 11 . The joint British Society of Gastroenterology (BSG) and European Society of Gastrointestinal Endoscopy (ESGE) guideline additionally states that low-risk polypectomies (i.e. cold snare polypectomies of lesions <10 mm), may also be safely performed with continuation of clopidogrel monotherapy 11 .

Following current guideline recommendations, patients using high-dose ATT, defined as ATT consisting of a direct oral anticoagulant (DOAC), a vitamin K antagonist (VKA), or dual antiplatelet therapy (DAPT), mostly temporarily discontinue ATT or change to single antiplatelet therapy (SAPT) around colonoscopy 7 8 9 10 11 . Consequently, insights regarding the risk of DPPB for patients on high-dose ATT are scarce. However, patients and clinicians could benefit from performing colonoscopy with continuation of high-dose ATT, as discontinuation increases the risk for thromboembolic events 12 13 14 15 , while discontinuation of ATT may also require consultation with other medical specialists and additional monitoring of coagulation parameters.

Although the evidence is limited, several recent studies have suggested that for low-risk polypectomies the continuation of high-dose ATT does not significantly increase the risk of DPPB 16 17 18 19 20 21 . In order to obtain additional insights into the safety and feasibility of (routine) continuation of high-dose ATT around colonoscopy, this study aimed to assess the incidence of DPPB and thromboembolic events for patients who either continued or discontinued different types of ATT around colonoscopy. Subgroup analyses were performed for routine diagnostic colonoscopies vs. colonoscopies after a positive fecal immunochemical test (FIT), and for procedures involving low-risk vs. high-risk polypectomies. Insights were used to evaluate the specific risks (probability of adverse events) and harms (actual negative outcomes or consequences resulting from adverse events) associated with continuation or discontinuation of high-dose ATT around colonoscopy.

Additionally, we aimed to obtain insights into the incidence of IPPB for patients on different types of ATT, the absolute and relative risks for occurrence of polypectomy-related bleeding (both IPPB and DPPB) for patients, procedures, and polyps with different characteristics, the number of patients for whom ATT should be discontinued to prevent one case of DPPB, and the extent to which high-dose ATT continuation results in the need for additional colonoscopies.

## Methods

### Setting and study design

This study analyzed data from Bergman Clinics, Amsterdam (center A) and Bergman Clinics, Bilthoven (center B). All data were structurally recorded as part of routine care. Patient-, colonoscopy-, and adverse event-related data were automatically extracted from electronic health records, endoscopy reporting systems, and the Dutch Registration of Complications in Endoscopy database 22 . Extracted data were analyzed to evaluate the incidence of polypectomy-related bleeding and thromboembolic events among patients who either continued or discontinued various types of ATT around colonoscopy.

The Institutional Review Board of the Amsterdam University Medical Center, Amsterdam (2023.0845) decided that formal revision according to the Medical Research Involving Human Subjects Act (WMO) was not required. Moreover, given the observational study design, the inclusion of a high number of patients, and the use of anonymized data, the requirement for obtaining informed consent was waived. Nonetheless, all patients receiving care at the participating centers were informed that their medical data could be (anonymously) used for research purposes and were given the opportunity to opt out. Data from patients who opted out were excluded from all data extractions.

### Study population

Data from all adult patients (≥18 years) who underwent colonoscopy at one of the study centers between January 2017 and January 2024 were analyzed. At both centers, information regarding the medical history and medication use of all patients was obtained during routine preprocedural outpatient consultations and was recorded within the local electronic health records (HiX; ChipSoft, Amsterdam, the Netherlands) using standardized reporting templates.

### Colonoscopy procedures

Colonoscopies were divided into diagnostic and therapeutic colonoscopies. Diagnostic colonoscopies were further categorized into two groups: those following a positive FIT result for the Dutch colorectal cancer screening program (i.e. FIT-positive colonoscopies), and all other colonoscopies conducted with a primary diagnostic purpose (i.e. routine diagnostic colonoscopies). Therapeutic colonoscopies encompassed procedures initiated for resection of previously detected polyps that could not be resected during the primary procedure (e.g. due to ATT use or time constraints). Although both centers ran outpatient diagnostic endoscopy clinics, therapeutic colonoscopies for purposes other than (advanced) polyp resection (e.g. treatment of acute gastrointestinal bleedings, dilation, stenting) were not performed. As this study specifically aimed to evaluate the safety and feasibility of (routine) continuation of (high-dose) ATT around diagnostic colonoscopies (i.e. procedures for which the presence of [advanced] neoplasia is not known in advance), therapeutic colonoscopies were excluded from the study analyses.

Colonoscopies were reported within local endoscopy reporting systems (Endobase; Olympus, Tokyo, Japan) using standardized reporting templates.

### Management of ATT

**Fig. 1:**
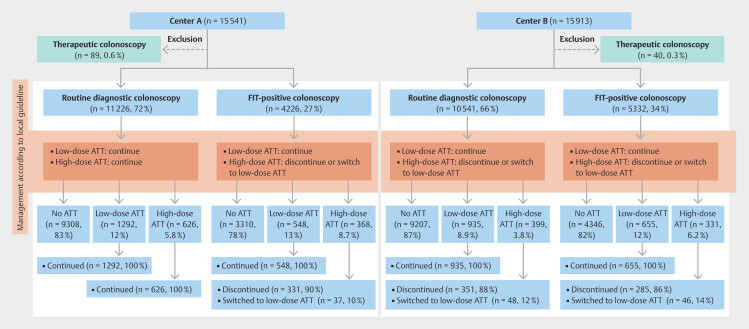
Flow chart depicting patient distribution by colonoscopy indication and antithrombotic therapy management according to local protocols. ATT, antithrombotic therapy; FIT, fecal immunochemical test.

The centers followed different protocols for the management of ATT around endoscopic procedures throughout the entire study period ( Fig. 1 ). Center A followed the hospital-wide guideline of the affiliated academic center, which was established in cooperation with the local vascular medicine department. For routine diagnostic colonoscopies in center A, all types of ATT were continued. Center B followed the Dutch national guideline, based on the BSG/ESGE recommendations 11 . For routine diagnostic colonoscopies in center B, ATT was only continued for patients on SAPT (any type) or a low-molecular-weight heparin (LMWH) at a prophylactic dose. For FIT-positive colonoscopies at either center, ATT other than SAPT or an LMWH at a prophylactic dose was discontinued (interrupted) or switched to SAPT (downgraded). Details regarding the timing and duration of ATT discontinuation, as determined by the local protocols, are reported in **Table 1s** in the online-only Supplementary material.

### Identification and reporting of adverse events

All adverse events occurring during the preparation for the colonoscopy, during the procedure itself, or within 30 days following the colonoscopy, were recorded. Post-procedural symptoms and adverse events were inquired about during routine telephone consultations scheduled within 1 month after each colonoscopy. In addition, patients were specifically requested to contact the clinic if they experienced any post-procedural symptoms or (potential) adverse events following the colonoscopy. Adverse events were recorded within both the local electronic health records and the Dutch Registration of Complications in Endoscopy database 22 . The severity of adverse events was graded using the AGREE classification 23 .

### Definition of study variables

IPPB was defined as abnormal or significant bleeding occurring immediately following polypectomy, typically necessitating therapeutic intervention (e.g. clipping) to establish hemostasis. The incidence of IPPB was based on documented occurrence of such bleeding in endoscopy reports. DPPB was defined as bleeding manifesting with hematochezia or melena from hours up to 30 days after a colonoscopy during which at least one polypectomy was performed. Thromboembolic events were defined as any event in which a blood clot led to a disrupted blood flow to any organ, thereby leading to tissue or organ damage (e.g. ischemic cardiovascular or cerebrovascular disease, pulmonary embolism, or deep vein thrombosis). Only DPPBs with a severity of AGREE grade I or higher were included for study analyses 23 , thereby excluding patients experiencing minor post-polypectomy bleeding without any clinical consequences.

Low-risk polypectomies were defined as resections of lesions <10 mm performed using a cold snare or biopsy forceps. All other polypectomies were classified as high risk. This distinction was made as a polyp size ≥10 mm and hot snare resection are recognized risk factors for DPPB 6 24 .

Low-dose ATT was defined as ATT consisting of SAPT (any type) or an LMWH at a prophylactic dose. High-dose ATT was defined as ATT consisting of an anticoagulant (VKA or DOAC), DAPT, or an LMWH at a therapeutic dose. For study analyses, patients who discontinued high-dose ATT were assigned to the “no ATT” group in cases where ATT was discontinued, or to the “low-dose ATT” group if ATT was switched to low-dose ATT.

The risk associated with continuation and discontinuation of ATT was defined as the probability of an adverse event occurrence within 30 days following a colonoscopy (cumulative incidence of DPPB and thromboembolic events), while the harm was defined as the actual negative outcomes or consequences resulting from the occurrence of adverse events (in terms of severity, duration, and impact on the patient).

### Study outcomes

The primary outcomes of the study were the incidence of DPPB and thromboembolic events following colonoscopies during which only low-risk polypectomies were performed, for patients who either continued or discontinued different types of ATT. Secondary outcomes were the incidence of DPPB after colonoscopies during which at least one high-risk polypectomy was performed, the incidence of IPPB, the differences in the absolute and relative risk for polypectomy-related bleeding (DPPB and IPPB) for specific subgroups of patients, procedures, and polyps, the number of patients who would need to discontinue ATT to prevent one case of DPPB, and the percentage of patients who (theoretically) required an additional colonoscopy due to continuation of high-dose ATT.

### Statistical analyses

Patient, colonoscopy, and polyp characteristics were described using descriptive statistics. Normality of data was checked using stem-and-leaf and QQ plots. DPPB incidence rates between different subgroups of patients were compared using Fisher’s exact tests. Subgroups were defined based on type of ATT (no vs. low dose vs. high-dose), as well as colonoscopy indication (routine diagnostic vs. FIT positive) and type of performed polypectomies (low-risk vs. high-risk).

Absolute risk differences (ARDs) and relative risks (RRs) for IPPB and DPPB were estimated using generalized linear models. For estimation of ARDs, models with an identity link function were used. For estimation of RRs, we ran the same model using a logistic link function. As calculation of confidence intervals in low-incidence settings may lead to invalid or unreliable results, the confidence intervals around the ARDs and RRs were calculated using bootstrapping with a 1000 iterations. Models used for estimation of ARDs were additionally used for estimation of the number needed to treat, defined as the number of patients who would need to discontinue ATT to prevent one case of DPPB.

Regression analyses involving DPPB were performed on a per-colonoscopy basis and with exclusion of colonoscopies during which no polypectomies were performed. To account for potential variation across centers, we included a dummy variable for center as a covariate in all analyses 25 . For analyses involving DPPB, we refrained from multivariable regression analyses including more than two variables, as the low DPPB incidence rates hampered validity of such multivariable models. Regression analyses involving IPPB were performed on a per-polyp basis. Multivariable analyses were performed using ATT use and polyp size, morphology, and location as independent variables, while also accounting for potential variation across locations by including center as a covariate 25 . Other variables were excluded due to either collinearity between variables or a low IPPB incidence rate within specific subgroups, compromising stability and validity of the models.

All analyses were performed using R version 4.4.1 (R Foundation for Statistical Computing, Vienna, Austria). *P* values <0.05, as determined using two-sided tests, were considered statistically significant.

## Results

A total of 31 325 diagnostic colonoscopies were performed in 27 714 unique patients during the study period ( Fig. 1 ). Patient and colonoscopy characteristics are shown in Table 1 . Colonoscopies were performed in 26 171 patients (83.5%) who were not using ATT, while 3430 (10.9%) and 1724 (5.5%) patients were on low- and high-dose ATT, respectively. For patients on high-dose ATT, ATT was continued around colonoscopy in 626 (36.3%), discontinued in 967 (56.1%), and switched to low-dose ATT in 131 (7.6%) patients ( Fig. 1 ). Only low-risk polypectomies were performed in 12 291 colonoscopies (39.2%), while at least one high-risk polypectomy was performed in 5100 procedures (16.3%). A total of 51 645 polyps were resected. Polyp detection and resection rates are reported in **Table 2s.** Characteristics of resected polyps are reported in **Table 3s** .

**Table:** **Table 1** Patient and colonoscopy characteristics, reported on a per-colonoscopy basis.

	Center A (n = 15 452), n (%)	Center B (n = 15 873), n (%)	All (n = 31 325), n (%)
Age, median (IQR), years	63 (56–70)	62 (55–69)	63 (56–70)
Sex, male , n (%)	8011 (51.8)	8074 (50.9)	16 085 (51.3)
ASA score, n (%)			
1	5648 (36.6)	5020 (31.6)	10 668 (34.1)
2	7542 (48.8)	9616 (60.6)	17 158 (54.8)
≥3	184 (1.2)	37 (0.2)	221 (0.7)
Unspecified	2078 (13.4)	1200 (7.6)	3278 (10.5)
Hypertension, n (%)			
No	10 255 (66.4)	11 073 (69.8)	21 328 (68.1)
Yes	4876 (31.6)	4267 (26.9)	9143 (29.2)
Unspecified	321 (2.1)	533 (3.4)	854 (2.7)
Cardiovascular disease ^1^ , n (%)			
No	13 018 (84.2)	13 398 (84.4)	26 416 (84.3)
Yes	1583 (10.2)	1618 (10.2)	3201 (10.2)
Unspecified	851 (5.5)	857 (5.4)	1708 (5.5)
Cerebrovascular disease			
No	14 460 (93.6)	14 838 (93.5)	29 298 (93.5)
Yes	676 (4.4)	505 (3.2)	1181 (3.8)
Unspecified	316 (2.0)	530 (3.3)	846 (2.7)
Antithrombotic therapy ^2^ , n (%)			
None	12 618 (81.7)	13 553 (85.4)	26 171 (83.5)
Low dose			
Single antiplatelet therapy ^3^	1835 (11.9)	1590 (10.0)	3425 (10.9)
LMWH, prophylactic dose	5 (<0.1)	0	5 (<0.1)
High dose			
Dual antiplatelet therapy ^3^	103 (0.7)	85 (0.5)	188 (0.6)
LMWH, therapeutic dose	1 (<0.1)	0	1 (<0.1)
VKA	189 (1.2)	138 (0.9)	327 (1.0)
VKA and antiplatelet therapy	3 (<0.1)	1 (<0.1)	4 (<0.1)
DOAC	680 (4.4)	498 (3.1)	1178 (3.8)
DOAC and antiplatelet therapy	18 (0.1)	8 (0.1)	26 (0.1)
Colonoscopy indication, n (%)			
Routine diagnostic colonoscopy			
Symptoms	6706 (43.4)	7751 (48.8)	14 457 (46.2)
Surveillance	3429 (22.2)	1866 (11.8)	5295 (16.9)
Familial risk	833 (5.4)	492 (3.1)	1325 (4.2)
Inflammatory bowel disease	55 (0.4)	75 (0.5)	130 (0.4)
Other	203 (1.3)	357 (2.2)	560 (1.8)
Colonoscopy after positive FIT within the CRCSP	4226 (27.3)	5332 (33.6)	9558 (30.5)
ASA, American Society of Anesthesiologists; CRCSP, colorectal cancer screening program; DOAC, direct oral anticoagulant; FIT, fecal immunochemical test; IQR, interquartile range; LMWH, low-molecular-weight heparin; VKA, vitamin K antagonist. Note: data were reported on a per-colonoscopy basis. As some patients underwent multiple colonoscopies, data of these patients were included multiple times. For each colonoscopy the patients’ characteristics at the time of colonoscopy were used for the study analyses. ^1^ Defined as a medical history with ischemic or vascular cardiac disease, atrial fibrillation, or valve replacement. ^2^ Reported numbers indicate the type of antithrombotic therapy patients were routinely using, rather than active antithrombotic therapy at the time of colonoscopy (antithrombotic therapy could have been discontinued or switched around colonoscopy, as reported in Fig. 1 ). ^3^ Antiplatelet therapy was defined as antithrombotic therapy involving one of the following agents: aspirin, clopidogrel, prasugrel, ticagrelor, or dipyridamole.			

### Delayed post-polypectomy bleedings

The overall incidence of DPPB after colonoscopies during which at least one polypectomy was performed was 58/17 391 (0.33%). The DPPB incidence rates for routine diagnostic and FIT-positive colonoscopies during which at least one polypectomy was performed were 30/10 191 (0.29%) and 28/7200 (0.39%), respectively ( Table 2 ). The DPPB incidence rate after colonoscopies during which only low-risk polypectomies were performed was 11/12 291 (0.09%), with specific incidence rates for patients on no, low-dose, and high-dose ATT of 6/10 363 (0.06%), 3/1585 (0.19%), and 2/343 (0.58%), respectively ( Table 3 ). The incidence rate of DPPB for patients discontinuing high-dose ATT was 4/967 (0.41%), while a DPPB occurred in 1/131 patients (0.76%) who switched high-dose ATT to low-dose ATT.

**Table:** **Table 2** Incidence of delayed post-polypectomy bleeding after diagnostic colonoscopies, stratified by center and type of active antithrombotic therapy at the time of colonoscopy.

	Colonoscopy indication and type of performed polypectomies ^1^					
	Routine diagnostic colonoscopies		FIT-positive colonoscopies		All	
	Low-risk	High-risk	Low-risk	High-risk	Low-risk	High-risk
**Center A**						
No ATT ^2^						
Total, n	3656	856	1775	1058	5431	1914
DPPB, n (%)	1 (0.03)	6 (0.70)	1 (0.06)	5 (0.47)	2 (0.04)	11 (0.57)
Low-dose ATT ^3^						
Total, n	609	116	290	167	899	283
DPPB, n %	1 (0.16)	2 (1.72)	0	2 (1.20)	1 (0.11)	4 (1.41)
High-dose ATT						
Total, n	343	191	NA	NA	343	19
DPPB, n %	2 (0.58)	1 (5.26)	NA	NA	2 (0.58)	1 (5.26)
**Center B**						
No ATT ^2^						
Total, n	3068	1006	1864	1518	4932	2524
DPPB, n%	4 (0.13)	8 (0.80)	0	17 (1.12)	4 (0.08)	25 (0.99)
Low-dose ATT ^3^						
Total, n	392	126	294	234	686	360
DPPB, n (%)	2 (0.51)	3 (2.38)	0	3 (1.28)	2 (0.29)	6 (1.67)
High-dose ATT						
Total, n	NA	NA	NA	NA	NA	NA
DPPB, n (%)	NA	NA	NA	NA	NA	NA
ATT, antithrombotic therapy; DPPB, delayed post-polypectomy bleeding; FIT, fecal immunochemical test; NA, not applicable. Note: 11 576 (n = 5627 center A; n = 5949 center B) routine diagnostic colonoscopies and 2358 (n = 936 center A; n = 1422 center B) FIT-positive colonoscopies during which no polypectomies were performed, were not reported. ^1^ Although not in line with the local protocol, high-risk polypectomy was performed in 19 patients on continued high-dose ATT, with a DPPB occurring after 1 (5.26%) of these procedures. Due to the small number of patients, no comparative analyses were performed involving this subgroup. ^2^ This group also includes colonoscopies performed in patients who discontinued direct oral anticoagulant (DOAC) or vitamin K antagonist (VKA) monotherapy (no active ATT at the time of colonoscopy). ^3^ This group also includes: (a) colonoscopies performed in patients receiving a combination of either a DOAC or VKA and an antiplatelet agent, who temporarily discontinued the DOAC or VKA but continued the antiplatelet agent around the colonoscopy; and (b) colonoscopies performed in patients on dual antiplatelet therapy who switched to single antiplatelet therapy around the colonoscopy.						

**Table:** **Table 3** Overall incidence and severity of delayed post-polypectomy bleeding after diagnostic colonoscopies (routine and following a positive fecal immunochemical test).

Type of ATT ^1^	Colonoscopies		Severity of DPPB according to AGREE classification, n (%) ^2^			
	Total, n	DPPB, n (%)	Grade I	Grade II	Grade IIIa	Grade IIIb/IV/V
Low-risk polyps resected						
No ATT	10 363	6 (0.06)	3 (50.00)	1 (16.67)	2 (33.33)	0
Low-dose ATT	1585	3 (0.19)	1 (33.33)	1 (33.33)	1 (33.33)	0
High-dose ATT	343	2 (0.58)	2 (100)	0	0	0
High-risk polyps resected						
No ATT	4438	36 (0.81)	17 (47.22)	2 (5.56)	17 (47.22)	0
Low-dose ATT	643	10 (1.56)	1 (10.00)	2 (20.00)	7 (70.00)	0
High-dose ATT	19	1 (5.26)	0	1 (100)	0	0
ATT, antithrombotic therapy; DPPB, delayed post-polypectomy bleeding. Note: 13,934 colonoscopies (n = 12 337 on no ATT, n = 1333 on low-dose ATT, n = 264 on high-dose ATT) during which no polypectomies were performed, were not reported. ^1^ Type of active antithrombotic therapy at the time of colonoscopy. ^2^ Grade I: adverse events requiring presentation at the emergency ward or hospital admission for <24 hours (without endoscopic, radiologic, or surgical intervention); grade II: adverse events requiring blood transfusion or hospital admission for >24 hours; grade IIIa: adverse events requiring endoscopic intervention.						

**Fig. 2:**
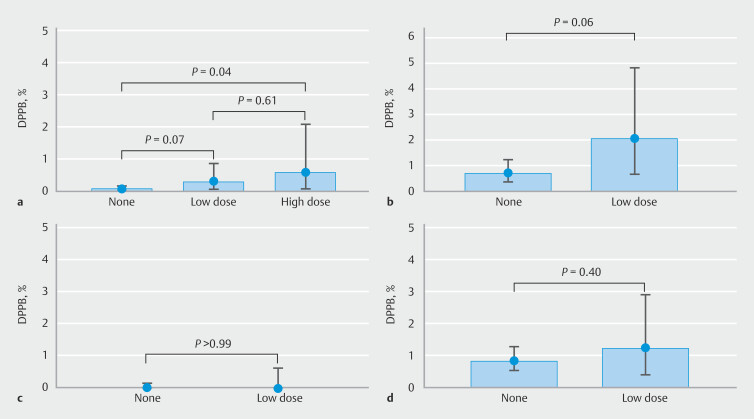
Incidence of delayed post-polypectomy bleeding (DPPB) for patients who underwent colonoscopy on different types of active antithrombotic therapy. The upper box limits and central black points indicate the DPPB incidence rate for each group. The error bars represent 95%CIs for the reported DPPB incidence rates. **a** Routine diagnostic colonoscopies during which only low-risk polypectomies were performed. **b** Routine diagnostic colonoscopies during which at least one high-risk polypectomy was performed. **c** Diagnostic colonoscopies following a positive fecal immunochemical test within the colorectal cancer screening program during which only low-risk polypectomies were performed. **d** Diagnostic colonoscopies following a positive fecal immunochemical test within the colorectal cancer screening program during which at least one high-risk polypectomy was performed.

For routine diagnostic colonoscopies during which only low-risk polypectomies were performed, DPPB incidence rates were similar for patients on high- and low-dose ATT (2/343 [0.58%, 95%CI 0.07 to 2.09] vs. 3/1001 [0.30%, 95%CI 0.06 to 0.87]; *P* = 0.61), as well as for patients on low-dose and no ATT (3/1001 [0.30%, 95%CI 0.06 to 0.87] vs. (5/6724 [0.07%, 95%CI 0.02 to 0.17]; *P* = 0.07) ( Fig. 2 **a** , **Table 4s** ). Although the incidence of DPPB for patients on high-dose ATT was significantly higher compared with patients on no ATT (2/343 [0.58%, 95%CI 0.07 to 2.09] vs. 5/6724 [0.07%, 95%CI 0.02 to 0.17]; *P* = 0.04) ( Fig. 2 **a** , **Table 4s** ), the incidence of DPPB was similar when comparing patients on high-dose ATT with the combined group of patients on either low-dose or no ATT (2/343 [0.58%, 95%CI 0.07 to 2.09] vs. 8/7725 [0.10%, 95%CI 0.04 to 0.20]; *P* = 0.07). Moreover, DPPB incidence rates were similar for patients on low-dose and no ATT after routine diagnostic colonoscopies during which at least one high-risk polypectomy was performed ( Fig. 2 **b** , **Table 4s** ), and for routine diagnostic colonoscopies involving only low-risk polypectomies in patients who either continued (center A) or discontinued (center B) high-dose ATT: 2/343 (0.58%, 95%CI 0.07 to 2.09) vs. 0/180 (0%, 95%CI 0.00 to 2.03; *P* = 0.55). For FIT-positive colonoscopies involving either only low-risk polypectomies or at least one high-risk polypectomy, the incidence of DPPB was also similar between patients on low-dose and no ATT ( Fig. 2 **c,d, Table 4s** ). Specific DPPB incidence rates for patients using (a combination of) different types of anticoagulants and antiplatelet agents are reported in **Table 5s.**

Assessment of ARDs showed a 0.36% (95%CI 0.03 to 0.66) and 0.67% (95%CI –0.15 to 1.72) higher risk of DPPB for patients on low- and high-dose ATT, respectively, compared with patients on no ATT. The corresponding number needed to treat (i.e. the estimated number of patients who would need to discontinue ATT to prevent one case of DPPB), was 281 for low-dose ATT and 150 for high-dose ATT. Colonoscopy characteristics associated with the highest increase in the absolute risk of DPPB were resection of at least one polyp ≥10 mm (0.83% [95%CI 0.57 to 1.13] increase), performance of at least one hot snare resection (0.95% [95%CI 0.61 to 1.13] increase), and occurrence of at least one IPPB (0.95% [95%CI 0.12 to 2.14] increase) ( Table 4 ).

**Table:** **Table 4** Regression analyses for assessment of absolute risk differences and relative risk of delayed post-polypectomy bleeding for patients and colonoscopies with various characteristics.

	Total colonoscopies, n	Colonoscopies with DPPB, n (%)	Absolute risk difference ^1^ , (95%CI), %	Relative risk ^1^ (95%CI)
Type of ATT ^2^				
None	14 801	41 (0.28)	Reference	Reference
Low dose	2228	14 (0.63)	0.36 (0.03 to 0.66)	2.32 (1.14 to 3.90)
High dose	362	3 (0.83)	0.67 (–0.15 to 1.72)	4.65 (0.00 to 12.54)
Use of ATT ^2^				
No	14 801	41 (0.28)	Reference	Reference
Yes	2590	17 (0.66)	0.41 (0.11 to 0.72)	2.37 (1.30 to 4.14)
Polyp size				
Only polyps <10 mm	12 669	13 (0.10)	Reference	Reference
≥1 polyp ≥10 mm	4722	45 (0.95)	0.83 (0.57 to 1.13)	8.88 (4.96 to 18.43)
Polyp morphology				
No pedunculated polyps	13 811	32 (0.23)	Reference	Reference
≥1 pedunculated polyps	3580	26 (0.73)	0.48 (0.21 to 0.80)	2.99 (1.75 to 5.13)
Polyp resection method				
Only CSP or BFP	13 633	17 (0.12)	Reference	Reference
≥1 HSP performed	3758	41 (1.09)	0.95 (0.61 to 1.13)	8.45 (4.86 to 16.03)
Number of polyps				
<3	9890	23 (0.23)	Reference	Reference
≥3	7501	35 (0.47)	0.21 (0.04 to 0.39)	2.01 (1.17 to 3.48)
IPPB				
No IPPB reported	16 914	52 (0.31)	Reference	Reference
≥1 IPPB reported	477	6 (1.26)	0.95 (0.12 to 2.14)	3.93 (1.26 to 8.63)
ATT, antithrombotic therapy; BFP, biopsy forceps polypectomy; CSP, cold snare polypectomy; DPPB, delayed post-polypectomy bleeding; HSP, hot snare polypectomy; IPPB, immediate post-polypectomy bleeding. Note: analyses were performed including data of 17 391 colonoscopies during which at least one polypectomy was performed. Colonoscopies during which no polypectomies (n = 13 934) were performed, were excluded. ^1^ To account for potential variation across centers, a dummy variable for center was included as a covariate in all analyses. ^2^ Active ATT at the time of colonoscopy.				

DPPBs occurring in patients on high-dose ATT seemed of comparable severity to DPPBs in patients on no or low-dose ATT: all reported DPPBs were grade I, II, or IIIa adverse events. All DPPBs occurring in patients on high-dose ATT were grade I or II adverse events ( Table 3 ).

### Thromboembolic events

Thromboembolic events occurred in 2/1098 patients (0.18%) who either discontinued (n = 967) or switched high-dose ATT to low-dose ATT (n = 131) ( **Table 6s** ). Both thromboembolic events were transient ischemic attacks occurring in FIT-positive patients who temporarily discontinued a DOAC prior to undergoing colonoscopy. Both patients were hospitalized for one night and recovered completely (grade II adverse events). No thromboembolic events occurred in patients on continued ATT.

### Immediate post-polypectomy bleedings

IPPB was reported for 519/51 645 polyps (1.00%). The IPPB incidence rates for polyps resected in patients on no, low-risk, and high-risk ATT were 427/43 248 (0.99%), 86/7453 (1.15%), and 6/944 (0.64%), respectively. Incidence rates of IPPB for polyps with different characteristics are shown in **Table 7s** . Multivariable regression revealed a distal polyp location (0.82% [95%CI 0.65 to 0.99] increase), polyp size ≥10 mm (1.27% [95%CI 0.86 to 1.68] increase), and pedunculated morphology (3.17% [95%CI 2.50 to 3.85] increase) as factors associated with a higher increase in the absolute risk of IPPB compared with the use of ATT (0.25% [95%CI 0.07 to 0.43] increase) ( **Table 8s** ).

### Continuation of ATT and need for additional colonoscopies

At center A, 11/626 patients (1.8%) on high-dose ATT and undergoing a routine diagnostic colonoscopy had to be rescheduled for colonoscopy as continuation of high-dose ATT hampered safe resection of advanced lesions (≥10 mm). Considering that high-risk polypectomies were performed in an additional 19 patients on continued high-dose ATT, mostly involving resection of polyps slightly exceeding 10 mm in size, strict adherence to the local protocol could theoretically have necessitated additional procedures in 30/626 patients (4.8%).

For center B, a total of 399 patients on high-dose ATT and undergoing a routine diagnostic colonoscopy discontinued ATT (n = 351, 88.0%) or switched to low-dose ATT (n = 48, 12.0%). Without making these changes, 22/399 patients (5.5%) would have required an additional colonoscopy due to the presence of advanced lesions. For 12 (54.6%) of these colonoscopies, the largest polyp was exactly 10 mm.

## Discussion

This study, involving a large multicenter colonoscopy cohort, showed that the incidence of DPPB after colonoscopies involving only cold snare polypectomies of lesions <10 mm was low, regardless of continuation of different types of ATT (up to 0.58% for patients on high-dose ATT). In addition, DPPB incidence rates were shown to be similar for patients on high- and low-dose ATT, and for patients on low-dose and no ATT. The incidence of DPPB was found to be higher for patients on high-dose ATT compared with patients on no ATT. However, the estimated ARD remained low (0.67%, 95%CI –0.15 to 1.72), with a high corresponding number of patients required to discontinue high-dose ATT to prevent one case of DPPB (n = 150). Additionally, the severity of DPPB did not appear to differ based on the type of active ATT.

Several preliminary studies have evaluated the risk of DPPB following low-risk polypectomies in patients on different ATT regimens. A recent meta-analysis reported a DPPB incidence rate after low-risk polypectomies for patients without ATT of 15/9508 (0.16%), while incidence rates of 6/336 (1.79%), 8/513 (1.56%), and 2/65 (3.08%) were reported for patients on (a) continued DOAC, VKA, or DAPT, respectively 5 . DPPB incidence rates as shown in our study (no ATT 6/10 363 [0.06%]; DOAC 2/234 [0.85%]; VKA 0/56 [0%]; DAPT: 0/44 [0%]) were all lower compared with the previously reported pooled estimates. These differences may be attributed to differences in characteristics of included patients and polyps, the use of divergent DPPB definitions, and a smaller number of patients in some of the subgroups in our study. However, most importantly, and irrespective of the underlying reasons for these differences, our study emphasizes that the risk of DPPB following low-risk polypectomies is low, even among patients on continued high-dose ATT 5 .

Some previous studies have also specifically compared DPPB incidence rates after low-risk polypectomies for patients on different ATT regimens 16 17 18 19 20 21 26 27 28 . Two studies compared incidence of DPPB in patients who either continued DAPT or switched DAPT to aspirin monotherapy, and reported no significant differences between the two groups 16 17 . A study comparing DPPB incidence rates for patients who either continued or discontinued a DOAC showed a significantly higher DPPB incidence rate in patients who continued ATT, although this study involved only a relatively small number of patients 26 . Most other studies compared DPPB incidence rates for patients on different types of ATT with patients not receiving ATT. Most of these studies showed no significant difference in DPPB incidence 18 19 20 21 , while in two studies the risk of DPPB was reported to be significantly higher for patients in the continued ATT group 27 28 . However, the generalizability of the results of these studies is limited, while the group of patients on continued ATT consisted of both patients on low- and high-dose ATT across all studies. The limited and inconclusive evidence regarding the safety of continuing high-dose ATT underscores the need for additional real-world data, which are provided in the current study.

The findings of our study have several clinical implications. Primarily, our results indicate that for colonoscopies involving only low-risk polypectomies, the incidence of DPPB is comparable for patients on high- and low-dose ATT. In particular, for patients on DAPT, or a combination of a DOAC or VKA with an antiplatelet agent, this finding may support the continuation of high-dose ATT instead of routinely switching to low-dose ATT, as is currently recommended 11 . Meanwhile, DPPB incidence rates were found to be higher for patients on high-dose ATT compared with patients on no ATT. Accordingly, it could be argued that routine discontinuation of high-dose ATT likely results in the most favorable clinical outcomes; however, routine discontinuation of high-dose ATT should be evaluated with consideration of the specific risks and harms associated with its discontinuation.

In terms of risks for patients discontinuing high-dose ATT or switching high-dose ATT to low-dose ATT, our study revealed incidence rates of DPPB and thromboembolic events of 0.46% (5/1098) and 0.18% (2/1098), respectively (summed risk: 0.64%). The corresponding incidence rates for patients continuing high-dose ATT were 0.58% and 0% (summed risk: 0.58%). Although continuation of high-dose ATT resulted in a higher absolute risk for DPPB (0.58% vs. 0.46%), the potential harms of discontinuing high-dose ATT may be greater. This primarily relates to the higher risk for thromboembolic events (0% vs. 0.18%), which are more likely to result in permanent (cerebrovascular or cardiovascular) damage compared with bleeding. In addition, as previously highlighted, an estimated 150 patients would require discontinuation of high-dose ATT to prevent one case of DPPB. Considering the high number of patients who would have to be exposed to an increased risk of thromboembolic events, this further supports the notion that the increased risk for thromboembolic events with discontinuation of high-dose ATT may not outweigh the (small) increased risk for DPPB. Finally, the incidence of thromboembolic events was relatively low in our study (0.18%) compared with preliminary studies (estimated at 0.9%–4.6%) 12 13 14 15 . This is likely attributable to the fact that both study centers ran outpatient diagnostic endoscopy clinics. Consequently, the patient population predominantly involved patients with a relatively low burden of comorbidities (American Society of Anesthesiologists score ≤2 for >99% of patients for which this information was documented). Therefore, our study likely underestimates the benefits of continuing high-dose ATT in preventing thromboembolic events for patients with higher (cardiovascular) comorbidity burdens.

Our study also highlights that DPPB incidence rates are comparable for patients on low-dose and no ATT. This supports current BSG/ESGE guideline recommendations stating that both aspirin and clopidogrel may be safely continued when low-risk polypectomies are performed 11 , especially when considering both the DPPB incidence rates for patients on continued low-dose ATT reported in our study (0.19%–1.56%), and thromboembolic event incidence rates with discontinuation of aspirin and clopidogrel reported in preliminary studies (0.3%–3.1%) 12 13 14 . In addition, we found similar DPPB incidence rates for patients on aspirin and other antiplatelet agents. This corroborates findings of the previously mentioned meta-analysis 5 and suggests that, especially for low-risk polypectomies, the risk of DPPB does not differ depending on the type of antiplatelet agent. These findings could prove useful for re-evaluation of guidelines that still distinguish between aspirin and other antiplatelet agents 7 8 9 10 . Moreover, the DPPB incidence rate was shown to be similar for patients on continued low-dose ATT and no ATT following colonoscopies in which high-risk polypectomies were performed. This even supports continuation of low-dose ATT when performing high-risk polypectomies. However, specific exploration of these results for different subgroups of polyps and resection techniques may be warranted.

In addition to lowering the risk for thromboembolic events, continuation of high-dose ATT could help to reduce the workload for healthcare practitioners. Consultation between medical specialists is often required to discuss the safety of ATT discontinuation. This approach may also reduce the burden for patients using agents that require dose adjustments and additional monitoring of coagulation parameters. Conversely, high-dose ATT continuation may prohibit safe excision of advanced (i.e. ≥10 mm) lesions, thereby potentially resulting in the need for a (preventable) repeat colonoscopy. These additional procedures are associated with considerable patient burden, as well as administrative burden and resource constraints for clinicians and endoscopy clinics. Meanwhile, our study revealed that only 11/15 452 (0.07%) colonoscopies in center A involved repeat procedures that could have been avoided through ATT discontinuation. Accordingly, the overall impact for clinicians and endoscopy clinics could be considered marginal.

In order to minimize the risk for a (preventable) repeat colonoscopy, continuation of high-dose ATT should mainly be considered for colonoscopies carrying a low risk of detecting advanced (≥10 mm) polyps. To estimate the likelihood of detecting advanced neoplasia during colonoscopy, endoscopists should consider patient-specific characteristics (e.g. sex, age) 29 , colonoscopy indication 30 31 , and the timing and findings of any prior colonoscopies 32 . Within the Dutch population, the prevalence of advanced polyps is higher for FIT-positive colonoscopies (30%–34% 33 34 ) than for colonoscopies with other diagnostic indications (bleeding 25%, symptoms 1%–17%, surveillance 5%–12%, familial risk 7%–10%, other 13%) 30 . This variation explains the different approaches for management of ATT for routine diagnostic and FIT-positive colonoscopies, as outlined in our study.

Finally, it is important to reflect on any (potential) differences between the two participating study centers. Both centers ran diagnostic outpatient endoscopy units and served comparable patient populations. Moreover, given their institutional affiliation, identical endoscopic equipment was employed at both sites. However, while both centers were staffed with experienced endoscopy nurses, there were differences in endoscopist experience: procedures in center A were performed by both supervised trainees (routine diagnostic colonoscopies only) and certified gastroenterologists, whereas all procedures at center B were conducted by certified gastroenterologists. This could be of clinical relevance, as one could hypothesize that less endoscopy experience may result in higher rates of polypectomy-related bleeding. Nonetheless, despite more experienced operators and more rigorous discontinuation of ATT, DPPB incidence was actually higher in center B ( **Table 2** ). This may be primarily attributable to the higher proportion of polyps ≥10 mm and hot snare resections at center B ( **Table 3s** ), for which our study emphasizes the relatively strong association with DPPB ( Table 4 ) 6 24 . As information regarding the endoscopist performing the procedure was lacking, our data did not allow for a per-endoscopist analysis of polypectomy-related bleeding rates. Accordingly, the extent to which bleeding rates were (additionally) affected by the experience of the endoscopist performing the procedure could not be analyzed. Such analyses may be considered for future research.

This study has several strengths. First, the different location-based protocols for management of ATT around colonoscopy in two mostly comparable outpatient endoscopy clinics provided a unique opportunity to compare incidence rates of DPPB and thromboembolic events. Moreover, data were structurally recorded as part of routine care, which omitted the need for retrospective data collection, thereby assuring high data quality. Second, a thorough evaluation of adverse events was performed through inquiry during routine (telephone) outpatient follow-up consultations, and all patients were carefully instructed to report any (potential) adverse events. In addition, the adverse events were derived from both local electronic health records and the Dutch Registration of Complications in Endoscopy database, thereby further reducing the chance of missing adverse events. Finally, we specifically reported both absolute and relative effect measures. Absolute effect measures better suit low-incidence settings compared with relative effect measures, and are, therefore, less prone to overestimate real-word impact 35 . Among others, this is illustrated by analyses involving polyp size in our study: while a polyp size ≥10 mm resulted in a nearly tenfold increase in the RR for DPPB, the absolute risk increase was only 0.83% (95%CI 0.57 to 1.13).

This study also has some limitations. First, the low incidence of DPPB limited the statistical power of comparative analyses and hindered multivariable analyses involving DPPB. However, considering that the study included over 31 000 colonoscopies, the lack of power in terms of DPPB incidence emphasizes the low risk of DPPB associated with low-risk polypectomies. Second, we used data from two Dutch outpatient diagnostic endoscopy clinics. Accordingly, factors such as the (local) Dutch guidelines and patient population may limit generalizability of our findings. Third, IPPBs were identified based on endoscopy reports. While identification and reporting of an IPPB is dependent on the endoscopist’s perception of what constitutes an abnormal or significant bleed, as well as the choice of whether or not therapeutic intervention is required to establish hemostasis, this may have affected IPPB incidence rates reported in our study. Meanwhile, the clinical relevance of this issue could be debated, as the clinical consequences of IPPB are mostly minor compared with DPPB. Finally, the majority of patients who experienced DPPBs did not undergo repeat colonoscopy at the time of active bleeding. In addition, whenever a repeat colonoscopy was performed, it was generally not performed at one of the study centers. As a result, our data did not allow for a per-polyp analysis of specific factors associated with DPPB occurrence.

To conclude, this study illustrated that in an outpatient diagnostic colonoscopy cohort, involving patients with relatively low comorbidity burdens, the incidence of DPPB following procedures in which only low-risk polypectomies were performed was very low, regardless of the use of various types of ATT. Moreover, for these procedures, the incidence of DPPB was found to be similar for patients on continued high- and low-dose ATT. For both colonoscopies during which low- and high-risk polypectomies were performed, the risk of DPPB was comparable for patients on continued low-dose and no ATT. Finally, the severity of DPPB did not appear to differ for patients with and without ATT, as well as for patients on either low- or high-dose ATT. Based on our study findings, we suggest routine continuation of high-dose ATT for colonoscopy indications carrying a low risk of detecting advanced polyps, while routine continuation of low-dose ATT should be considered for all colonoscopies. This may improve clinical outcomes and ease management of ATT around colonoscopies.

## Supplementary Material

Supplementary Material Zusatzmaterial
