# Supplementary material for: Delayed post-polypectomy bleeding following cold snare polypectomy of lesions <10 mm in patients on high-dose antithrombotic therapy: insights from a Dutch colonoscopy cohort
Source: Endoscopy. 2025 Nov 20;58(4):384–96. doi: 10.1055/a-2721-3151 (PMC13093077; doi:10.1055/a-2721-3151)
Supplement: Supplementary file 1 — Supplementary Material [file 10-1055-a-2721-3151_27504697.pdf]

Supplementary material

Delayed post-polypectomy bleeding following cold snare polypectomy of lesions <10 mm in patients on high-dose antithrombotic therapy: insights from a Dutch colonoscopy cohort

Querijn N. E. van Bokhorst, Sophie te Marvelde, Jos W. Borkent, Paul Fockens, Evelien Dekker, Manon van der Vlugt

Table 1s Timing and duration of antithrombotic therapy discontinuation (applicable for both study centers).

Type of antithrombotic agent(s)	Renal function	Management: Discontinuation	Management: Continuation
**DOAC**			
• Apixaban<br>• Edoxaban<br>• Rivaroxaban<br>• LMWH (therapeutic dosage)	*NA*	▪ Last dose on day -2	▪ First dose at day +1*†
• Dabigatran	eGFR ≥50 ml/min	▪ Last dose on day -2	▪ First dose at day +1*†
	eGFR <50 ml/min	▪ Last dose on day -3	▪ First dose at day +1*†
**VKA**			
• Acenocoumarol	*NA*	▪ Last dose on day -3<br>▪ Check INR on day -1 or 0:<br>- Routine diagnostic colonoscopies: aim for INR ≤3.0<br>- FIT-positive colonoscopies and therapeutic colonoscopies: aim for INR ≤1.5	▪ First dose at day +1*†
• Phenprocoumon	*NA*	▪ Last dose on day -5<br>▪ Check INR on day -1 or 0:<br>- Routine diagnostic colonoscopies: aim for INR ≤3.0<br>- FIT-positive colonoscopies and therapeutic colonoscopies: aim for INR ≤1.5	▪ First dose at day +1*†
**DAPT**			
• Aspirin + dipyridamole	*NA*	▪ Continue aspirin, last dose of dipyridamole >24 hours before procedure	▪ First dose of dipyridamole 24-48 hours after procedure
• Aspirin + clopidogrel / prasugrel / ticagrelor‡	*NA*	▪ Continue aspirin, discontinue other agent<br>- Clopidogrel: last dose between day -7 and day -5<br>- Prasugrel: last dose between day -7 and day -5<br>- Ticagrelor: last dose between day -7 and day -5	▪ First dose of discontinued agent at day +1
**Multiple agents (other than DAPT)**			
• DOAC + antiplatelet agent	*NA*	▪ Continue antiplatelet agent, discontinue DOAC following recommendations listed within section 'DOAC',	▪ Restart antiplatelet agent following recommendations listed within section 'DAPT'
• VKA + antiplatelet agent	*NA*	▪ Continue antiplatelet agent, discontinue VKA following recommendations listed within section 'DOAC'	▪ Restart antiplatelet agent following recommendations listed section 'DAPT'

NA, not applicable; DOAC, direct oral anticoagulant; LMWH, low-molecular-weight heparin; eGFR, estimated glomerular filtration rate; VKA, vitamin K antagonist; INR, International Normalized Ratio; DAPT, dual antiplatelet therapy. *For procedures with a very high bleeding risk (endoscopic mucosal resection of lesions ≥20 mm in the right-sided colon), restart of antithrombotic therapy may be postponed to anywhere between day +2 to day +7. The timing of the restart of antithrombotic therapy is based on the discretion of the endoscopist, considering the estimated bleeding risk of the lesion and the patient's cardiovascular risk profile; †First dose during the evening; ‡Always consult neurologist, cardiologist or vascular physician to discuss safety of discontinuing dual antiplatelet therapy.

Table 2s Polyp detection and resection rates.

	Center A		Center B		All	
	Routine diagnostic colonoscopies, n (%)	FIT-positive colonoscopies, n (%)	Routine diagnostic colonoscopies, n (%)	FIT-positive colonoscopies, n (%)	Routine diagnostic colonoscopies, n (%)	FIT-positive colonoscopies, n (%)
Total number of colonoscopies	11,226	4,226	10,541	5,332	21,767	9,558
Total number of detected polyps	14,859	13,554	11,773	13,650	26,632	27,204
Median (IQR) number of polyps per colonoscopy	1 (0-2)	2 (1-5)	0 (0-2)	2 (0-2)	0 (0-2)	2 (1-4)
Mean (±SD) number of polyps per colonoscopy	1.32 (±2.06)	3.21 (±3.26)	1.12 (±1.84)	2.56 (±2.66)	1.22 (±1.96)	2.85 (±2.96)
Total number of resected polyps	14,193	12,924	11,348	13,180	25,541	26,104
Median (IQR) number of polyps per colonoscopy	0 (0-2)	2 (1-4)	0 (0-1)	2 (0-4)	0 (0-2)	2 (1-4)
Mean (±SD) number of polyps per colonoscopy	1.26 (±2.01)	3.06 (±3.18)	1.08 (±1.81)	2.47 (±2.64)	1.17 (±1.92)	2.73 (±2.91)
Total number of colonoscopies with ≥1 polyp detected	5,805 (52)	3,384 (80)	4,771 (45)	4,027 (76)	10,576 (49)	7,411 (78)
Median (IQR) number of polyps per colonoscopy	2 (1-3)	3 (2-5)	2 (1-3)	3 (1-5)	2 (1-3)	3 (2-5)
Mean (±SD) number of polyps per colonoscopy	2.56 (±2.24)	4.01 (±3.17)	2.47 (±2.03)	3.39 (±2.57)	2.52 (±2.15)	3.67 (±2.88)
Total number of colonoscopies with ≥1 polyp resected	5,599 (50)	3,290 (78)	4,592 (44)	3,910 (73)	10,191 (47)	7,200 (75)
Median (IQR) number of polyps per colonoscopy	2 (1-3)	3 (2-5)	2 (1-3)	3 (1-5)	2 (1-3)	3 (1-5)
Mean (±SD) number of polyps per colonoscopy	2.53 (±2.21)	3.93 (±3.09)	2.47 (±2.02)	3.37 (±2.55)	2.51 (±2.13)	3.63 (±2.82)

FIT, fecal immunochemical test; IQR, interquartile range; SD, standard deviation. Note: while the numbers of detected and resected polyps mostly concerned non-normally distributed data (right-skewed), the numbers of detected and resected polyps are primarily reported as medians with IQRs. As mean numbers of detected and resected polyps (with SDs) may allow for easier interpretation, both the median and mean numbers of detected and resected polyps are reported. However, considering the non-normal distribution of data, means should be interpreted with caution.

Table 3s Characteristics of resected polyps.

	Center A		Center B		All	
	Routine diagnostic colonoscopies, n (%)	FIT-positive colonoscopies, n (%)	Routine diagnostic colonoscopies, n (%)	FIT-positive colonoscopies, n (%)	Routine diagnostic colonoscopies, n (%)	FIT-positive colonoscopies, n (%)
**Total number of resected polyps**	**14,193**	**12,924**	**11,348**	**13,180**	**25,541**	**26,104**
Location*						
Proximal colon	9,022 (64)	7,860 (61)	6,867 (61)	7,542 (57)	15,889 (62)	15,402 (69)
Distal colon	5,163 (36)	5,063 (39)	4,478 (39)	5,638 (43)	9,641 (38)	10,701 (41)
Other or unspecified	8 (<1)	1 (<1)	3 (<1)	0	11 (<1)	1 (<1)
Size						
<10 mm	13,140 (93)	11,380 (88)	10,019 (88)	10,849 (82)	23,159 (91)	22,229 (85)
≥10 mm	1,050 (7.4)	1,544 (12)	1,327 (12)	2,331 (18)	2,377 (9.3)	3,875 (15)
Unspecified	3 (<1)	0	2 (<1)	0	5 (<1)	0
Morphology						
Non-pedunculated	13,449 (95)	11,678 (90)	10,453 (92)	11,571 (88)	23,902 (94)	23,249 (89)
Pedunculated	733 (5.2)	1,246 (9.6)	845 (7.4)	1,609 (12)	1,578 (6.2)	2,855 (11)
Unspecified	11 (<1)	0	50 (<1)	0	61 (<1)	0
Pre-resection treatment						
None	13,440 (95)	11,588 (90)	10,422 (92)	11,438 (87)	23,862 (93)	23,026 (88)
Clips on stalk (pedunculated polyps)	0	7 (<1)	17 (<1)	35 (<1)	17 (<1)	42 (<1)
Lifting (with epinephrine)	200 (1.4)	404 (3.1)	424 (3.7)	1,000 (7.6)	624 (2.4)	1,404 (5.4)
Lifting (without epinephrine)	505 (3.6)	748 (5.8)	343 (3.0)	496 (3.8)	848 (3.3)	1,244 (4.8)
Endoloop	48 (<1)	177 (1.4)	142 (1.3)	211 (1.6)	190 (<1)	388 (1.5)
Resection technique						
Cold snare	13,222 (93)	11,399 (88)	10,363 (91)	11,516 (87)	23,585 (92)	22,915 (88)
Hot snare	901 (6.3)	1,494 (12)	919 (8.1)	1,637 (12)	1,820 (7.1)	3,131 (12)
Biopsy forceps	70 (<1)	31 (<1)	66 (<1)	27 (<1)	136 (<1)	58 (<1)
Post-resection treatment						
None	14,118 (99)	12,782 (99)	11,200 (99)	12,888 (98)	25,318 (99)	25,670 (98)
Clip placement†	75 (<1)	142 (1.1)	136 (1.2)	263 (2.0)	211 (<1)	405 (1.6)
Coagulation	0	0	1 (<1)	0	1 (<1)	0
Epinephrine injection	0	0	11 (<1)	29 (0.2)	11 (<1)	29 (0.1)

FIT, fecal immunochemical test; *The distal colon consists of the cecum, ascending colon, hepatic flexure, transverse colon, transverse colon and splenic flexure; the proximal colon consists of the descending colon, sigmoid colon and rectum; †Prophylactic or therapeutic clip placement.

Table 4s Comparison of the overall incidence of delayed post-polypectomy bleedings, stratified by colonoscopy indication and type of antithrombotic therapy.

	Type of antithrombotic therapy*	DPPB	No DPPB	Percentage (95% CI)	P-value
Routine diagnostic colonoscopies, only low-risk polypectomies	None	5	6,719	0.07 (0.02-0.17)	0.07
	Low-risk	3	998	0.30 (0.06-0.87)	
	None	5	6,719	0.07 (0.02-0.17)	0.04
	High-risk	2	341	0.58 (0.07-2.09)	
	Low-risk	3	998	0.30 (0.06-0.87)	0.61
	High-risk	2	341	0.58 (0.07-2.09)	
Routine diagnostic colonoscopies, at least one high-risk polypectomy	None	14	1,848	0.76 (0.41-1.27)	0.06
	Low-risk	5	237	2.11 (0.69-4.85)	
FIT-positive colonoscopies, only low-risk polypectomies	None	1	3,638	0.03 (0.00-0.15)	1.00
	Low-risk	0	584	0.00 (0.00-0.63)	
FIT-positive colonoscopies, at least one high-risk polypectomy	None	22	2,554	0.86 (0.54-1.30)	0.40
	Low-risk	5	396	1.26 (0.41-2.92)	

DPPB, delayed post-polypectomy bleeding; CI, confidence interval; FIT, fecal immunochemical test. *Type of active antithrombotic therapy at the time of colonoscopy, as defined in **Figure 1**. Note: the group of patients for whom high-dose antithrombotic therapy was continued, and in whom at least one high-risk polypectomy was performed (n = 19, occurrence of a single DPPB), is not reported in this table.

Table 5s Incidence of delayed post-polypectomy bleedings after colonoscopies during which at least one polypectomy is performed, stratified by type of active antithrombotic agent(s) at the time of colonoscopy.

**Low-risk antithrombotic therapy**																		
	**Aspirin, n**			**Clopidogrel, n**			**Prasugrel, n**			**Ticagrelor, n**			**Dipyridamole, n**			**LMWH prophylactic, n**		
	**Total**	**DPPBs**	**%**	**Total**	**DPPBs**	**%**	**Total**	**DPPBs**	**%**	**Total**	**DPPBs**	**%**	**Total**	**DPPB**	**%**	**Total**	**DPPBs**	**%**
**Low-risk polyps resected**	1,154	3	0.26	416	1	0.24	0	0	0	2	0	0	9	0	0	2	0	0
**High-risk polyps resected**	480	3	0.63	160	7	4.38	2	0	0	0	0	0	2	0	0	0	0	0

**High-risk antithrombotic therapy**																		
	**VKA, n**			**DOAC**			**VKA + APA**			**DOAC + APA**			**DAPT**			**LMWH therapeutic, n**		
	**Total**	**DPPBs**	**%**	**Total**	**DPPBs**	**%**	**Total**	**DPPBs**	**%**	**Total**	**DPPBs**	**%**	**Total**	**DPPBs**	**%**	**Total**	**DPPBs**	**%**
**Low-risk polyps resected**	56	0	0	234	2	0.85	1	0	0	7	0	0	44	0	0	1	0	0
**High-risk polyps resected**	6	0	0	10	1	10.00	0	0	0	0	0	0	3	0	0	0	0	0

DPPBs, delayed post-polypectomy bleedings; LMWH, low-molecular-weight heparin; VKA, vitamin K antagonist; DOAC, direct oral anticoagulant; APA, antiplatelet agent; DAPT, double antiplatelet therapy. Note: 41 DPPBs that occurred in patients without antithrombotic therapy (n = 14,801 colonoscopies during which at least one polyp was resected) were not reported.

Table 6s Incidence of thromboembolic events for patients discontinuing high-dose antithrombotic therapy or switching high-dose antithrombotic therapy to low-dose antithrombotic therapy around colonoscopy.

Type of ATT	Number of patients discontinuing ATT, n	TE incidence rate, n (%)
VKA	199	0 (0)
VKA + antiplatelet agent*	2	0 (0)
DOAC	768	2 (0.26)
DOAC + antiplatelet agent*	13	0 (0)
DAPT*	116	0 (0)

ATT, antithrombotic therapy; TE, thromboembolic event; VKA, vitamin K antagonist; DOAC, direct oral anticoagulant; DAPT, dual antiplatelet therapy; *In accordance with local protocols, antithrombotic therapy in these patients was changed to single antiplatelet therapy (discontinuation of VKA or DOAC, continuation of antiplatelet agent).

Table 7s Characteristics of polyps for which an immediate post-polypectomy bleeding was reported.

	Center A						Center B						All					
	Routine diagnostic colonoscopies, n			FIT-positive colonoscopies, n			Routine diagnostic colonoscopies, n			FIT-positive colonoscopies, n			Routine diagnostic colonoscopies, n			FIT-positive colonoscopies, n		
	Total	IPPB	%	Total	IPPB	%	Total	IPPB	%	Total	IPPB	%	Total	IPPB	%	Total	IPPB	%
**Total**	14,193	76	0.54	12,924	145	1.12	11,348	98	0.86	13,180	200	1.52	25,541	174	0.68	26,104	345	1.32
**Location***																		
**Proximal colon**	5,163	45	0.87	5,063	98	1.94	4,478	77	1.72	5,638	180	3.19	9,641	122	1.27	10,701	278	2.60
**Distal colon**	9,022	30	0.33	7,860	47	0.60	6,867	21	0.31	7,542	20	0.27	15,889	51	0.32	15,402	67	0.44
**Other or unspecified**	8	1	12.50	1	0	0	3	0	0	0	0	0	11	1	9.09	1	0	0
**Size**																		
**<10 mm**	13,140	53	0.40	11,380	84	0.74	10,019	68	0.68	10,849	110	1.01	23,159	121	0.52	22,229	194	0.87
**≥10 mm**	1,050	23	2.19	1,544	61	3.95	1,327	30	2.26	2,331	90	3.86	2,377	53	2.23	3,875	151	3.90
**Unspecified**	3	0	0	0	0		2	0	0	0	0	0	5	0	0	0	0	0
**Morphology**																		
**Non-pedunculated**	13,449	53	0.39	11,678	75	0.64	10,453	69	0.66	11,571	111	0.96	23,902	122	0.51	23,249	186	0.80
**Pedunculated**	733	23	3.14	1,246	70	5.62	845	29	3.43	1,609	89	5.53	1,578	52	3.30	2,855	159	5.57
**Unspecified**	11	0	0	0	0		50	0	0	0	0	0	61	0	0	0	0	0
**Pre-resection treatment**																		
**None**	13,440	0	0	11,588	144	1.24	10,422	0	0	11,438	196	1.71	23,862	0	0	23,026	340	1.48
**Clips on stalk**	0	0	0	7	0	0	17	2	11.76	35	3	8.57	17	2	11.76	42	3	7.14
**Lifting (with epinephrine)**	200	0	0	404	0	0	424	0	0	1,000	1	0.10	624	0	0	1,404	1	0.07
**Lifting (without epinephrine)**	505	0	0	748	1	0.13	343	0	0	496	0	0	848	0	0	1,244	1	0.08
**Endoloop**	48	0	0	177	0	0	142	0	0	211	0	0	190	0	0	388	0	0
**Resection technique**																		
**Cold snare**	13,222	159	1.20	11,399	103	0.90	10,363	87	0.84	11,516	159	1.38	23,585	246	1.04	22,915	262	1.14
**Hot snare**	901	41	4.55	1,494	42	2.81	919	10	1.09	1,637	41	2.50	1,820	51	2.80	3,131	83	2.65
**Biopsy forceps**	70	0	0	31	0	0	66	1	1.52	27	0	0	136	1	0.74	58	0	0

FIT, fecal immunochemical test; IPPB, immediate post-polypectomy bleeding; *The distal colon consists of the cecum, ascending colon, hepatic flexure, transverse colon, transverse colon and splenic flexure; the proximal colon consists of the descending colon, sigmoid colon and rectum.

Table 8s Univariable regression analyses assessing the association between different patient- and polyp-related factors and the risk on immediate post-polypectomy bleeding.

		Total number of polyps	Number of polyps with IPPB (%)	Univariable analysis: Absolute risk difference, % (95% CI)	Univariable analysis: Relative risk (95% CI)	Multivariable analysis*: Absolute risk difference, % (95% CI)	Multivariable analysis*: Relative risk (95% CI)
**Type of ATT**	None	43,248	427 (0.99)	Reference	Reference	Reference	Reference
	Low-risk	7,453	86 (1.15)	0.17 (-0.09 to 0.43)	1.17 (0.91 to 1.46)	*NA*†	*NA*†
	High-risk	944	6 (0.64)	-0.35 (-0.84 to 0.24)	0.64 (0.18 to 1.25)	*NA*†	*NA*†
**Use of ATT**	No	43,248	427 (0.99)	Reference	Reference	Reference	Reference
	Yes	8,397	92 (1.10)	0.11 (-0.14 to 0.36)	1.11 (0.87 to 1.38)	0.25 (0.07 to 0.43)	1.30 (1.04 to 1.62)
**Polyp location**	Proximal	31,291	118 (0.38)	Reference	Reference	Reference	Reference
	Distal	20,342	400 (1.97)	1.59 (1.39 to 1.79)	5.21 (4.27 to 6.47)	0.82 (0.65 to 0.99)	3.43 (2.77 to 4.29)
**Polyp size**	<10	45,388	315 (0.69)	Reference	Reference	Reference	Reference
	≥10 mm	6,252	204 (3.26)	2.57 (2.13 to 3.05)	4.70 (3.95 to 5.70)	1.27 (0.86 to 1.68)	1.88 (1.47 to 2.38)
**Polyp morphology**	Non-pedunculated	47,151	308 (0.65)	Reference	Reference	Reference	Reference
	Pedunculated	4,433	211 (4.76)	4.11 (3.54 to 4.77)	7.29 (6.18 to 8.68)	3.17 (2.50 to 3.85)	2.97 (2.33 to 3.78)
**Polyp pre-treatment‡**	Yes	2,665	5 (0.19)	Reference	Reference	Reference	Reference
	No	48,980	514 (1.05)	0.86 (0.67 to 1.04)	5.59 (2.96 to 27.98)	*NA*†	*NA*†
**Polyp resection method**	CSP or BFP	46,694	404 (0.87)	Reference	Reference	Reference	Reference
	HSP	4,951	115 (2.32)	1.46 (1.04 to 1.90)	2.68 (2.16 to 3.26)	*NA*§	*NA*§

IPPB, immediate post-polypectomy bleeding; CI, confidence interval; ATT, antithrombotic therapy; NA, not available; CSP, cold snare polypectomy; BFP, biopsy forceps polypectomy; HSP, hot snare polypectomy; ***To account for potential variation across centers, a dummy variable for center was included as a covariate in all analyses; †Variable not included in multivariable analyses due to low incidence rate of IPPBs in specific subgroups, thereby compromising the validity of the multivariable model. ‡Pre-treatment was defined as prophylactic clipping of the stalk, placement of an endoloop, or lifting using a solution containing epinephrine; §Variable not included in multivariable analyses due to high collinearity with polyp size (Pearson's correlation coefficient = 0.72) and polyp morphology (Pearson's correlation coefficient = 0.56). Note: in case of missing data, polyps were excluded from the analyses.
